# Supplementary figures and images for: Multi-class rice seed recognition based on deep space and channel residual network combined with double attention mechanism
Source: PLoS One. 2025 May 16;20(5):e0322699. doi: 10.1371/journal.pone.0322699 (PMC12083804; doi:10.1371/journal.pone.0322699)

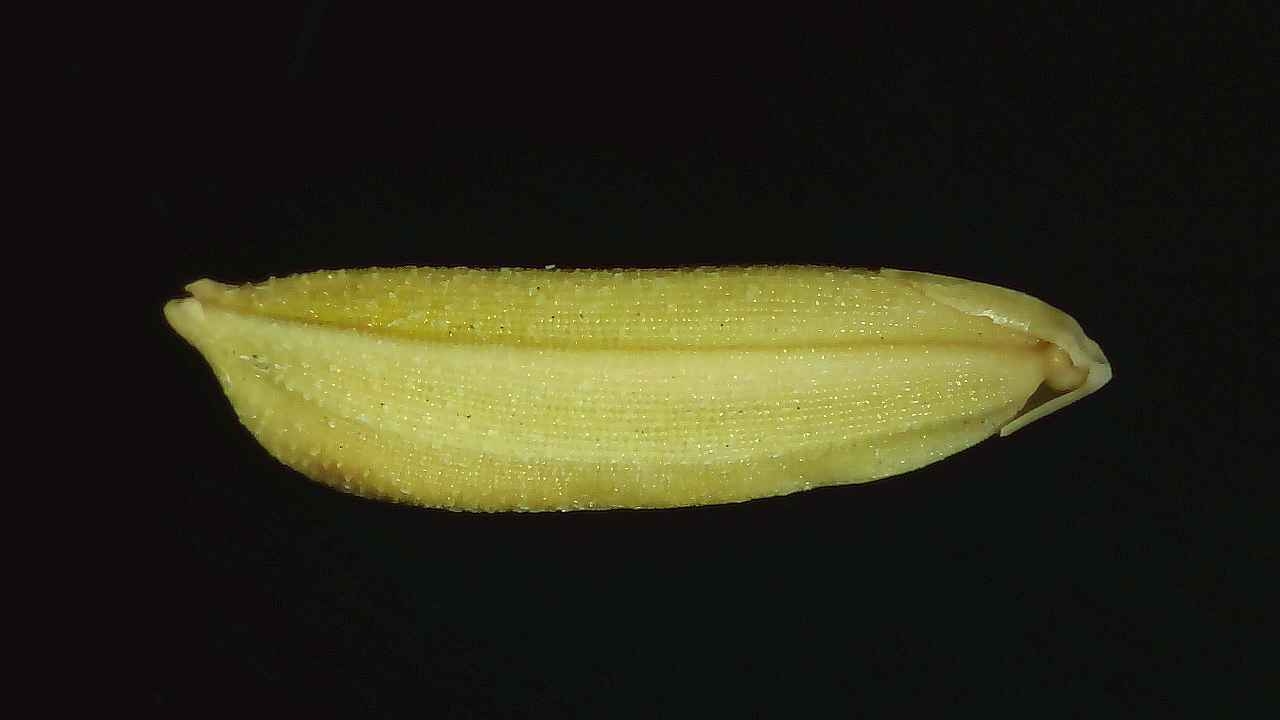

Supplement: S1 File — (ZIP) [file pone.0322699.s001.zip › coded file/chuanzhongyou6099(1).jpg]
