# Supplementary material for: Multi-class rice seed recognition based on deep space and channel residual network combined with double attention mechanism
Source: PLoS One. 2025 May 16;20(5):e0322699. doi: 10.1371/journal.pone.0322699 (PMC12083804; doi:10.1371/journal.pone.0322699)
Supplement: Attachment — Submitted filename: review.docx [file pone.0322699.s002.docx]

The manuscript presents a novel deep learning model, RSCD-Net, for the classification of 36 rice seed varieties, addressing challenges in fine-grained image recognition. The study demonstrates innovation through the introduction of the Space and Channel Residual Block (SCR-Block) and Double Attention Mechanism (A2Net). These features improve the classification accuracy and efficiency, surpassing baseline models like ResNet50 and others.

**Strengths:**

1. **Novelty**: The RSCD-Net model incorporates innovative mechanisms (SCR-Block and A2Net) that address inter-class variability and redundancy in feature extraction.
2. **Dataset**: A self-collected dataset of 36 rice varieties is commendable, as such datasets are rare and critical for agricultural research.
3. **Performance**: The model achieves superior accuracy (82.61%) compared to established networks, with detailed experimental validations.
4. **Scientific Rigor**: Ablation studies and comparisons with multiple benchmarks provide a robust evaluation of the proposed model's effectiveness.
5. **Practical Relevance**: The study has significant implications for agricultural research and industrial applications, especially in seed sorting and quality assessment.

**Weaknesses:**

1. **Dataset Limitations**: While the dataset is unique, the sample size per variety (240 for training) is relatively small for deep learning, potentially limiting the model's generalizability.
2. **Comparative Analysis**: Although the model outperforms ResNet50 and similar architectures, a comparison with newer state-of-the-art methods like Vision Transformers (ViT) would strengthen the claim of superiority.
3. **Reproducibility**: The manuscript does not provide complete details for dataset access or pretrained model weights, which are essential for reproducibility.
4. **Computational Efficiency**: While RSCD-Net reduces redundancy, its computational cost compared to lightweight models like MobileNetV3 remains high, which may limit deployment in resource-constrained environments.
5. **Clarity in Writing**: The manuscript has some complex descriptions, especially in the Materials and Methods section, which could be streamlined for better understanding.

**Recommendations:**

1. **Expand Dataset**: Consider increasing the dataset size or incorporating external datasets for better generalization.
2. **Broader Comparisons**: Include experiments with state-of-the-art models like ViT or Swin Transformers for a more comprehensive evaluation.
3. **Detailed Ablation**: Provide additional insights into the contributions of SCR-Block and A2Net individually through detailed visualizations.
4. **Provide Resources**: Share dataset links, code repositories, or pretrained models to enhance reproducibility.
5. **Improve Clarity**: Simplify complex sections and add more diagrams or flowcharts to explain model architecture and methodology.
